# Evaluating transient risk factors for avian influenza outbreaks in Canada using case-crossover study and machine learning

**DOI:** 10.1016/j.psj.2026.107364

**Published:** 2026-07-07

**Authors:** Iman Sekhavati, Rozita Dara, Shayan Sharif, Amir A. Aliabadi

**Affiliations:** a Department of Mechanical Engineering, College of Engineering, University of Guelph, Guelph, Ontario, Canada; b Department of Computer Science, College of Computational, Mathematical and Physical Sciences, University of Guelph, Guelph, Ontario, Canada; c Department of Pathobiology, Ontario Veterinary College, University of Guelph, Guelph, Ontario, Canada

**Keywords:** Canadian poultry, Highly Pathogenic Avian Influenza (HPAI), Outbreaks, Logistic regression, Risk factors

## Abstract

Highly Pathogenic Avian Influenza (HPAI) poses a severe biological and economic threat to the Canadian poultry industry. The transient and day-to-day meteorological and regional triggers remain poorly quantified due to spatial confounding in traditional epidemiological models. To isolate these triggers, this study utilizes a time-stratified case-crossover design, integrating a veterinary surveillance dataset of infected premises (2022–2024) with ERA5 meteorological reanalysis data. We employ the conditional difference method optimized with Ridge (L2) regularization. The national model demonstrated discriminative ability (ROC-AUC = 0.978; Brier score = 0.060). The analysis identified local proximity to an active outbreak as the strongest statistical predictor, with patterns consistent with wind-mediated transmission amplified by high wind speeds and relative humidity. A provincial-level stratification revealed that transmission mechanics are geographically distinct. In dense agricultural zones, extreme farm proximity dictates an effect that overpowers ambient weather factors. In dispersed geography of the Prairies, meteorological factors dominate infection triggers. These findings suggest that mitigation interventions must be tailored to regional factors.

## Highlights

• A logistic regression model is used to analyze outbreak reports across poultry facilities in Canada.
• The statistical model is highly accurate in identifying risk factors for influenza outbreaks.
• Risk factors are region-specific for each Canadian province.
• Among risk factors are farm density, outbreak status nearby, and meteorological conditions.
• The findings can inform policy makers to devise effective mitigation measures against outbreaks.

## Introduction

Highly Pathogenic Avian Influenza (HPAI), particularly the H5N1 sub-type, represents a significant and major global threat to both animal and public health, as well as to the poultry industry and economies (Biswas et al., 2014, Chen et al., 2022, Gkrinia et al., 2025). Since its emergence in 1996 and rapid spread in 2003, HPAI has caused pandemics in poultry and wild birds, affecting over 60 countries. The disease is devastating, with H5N1 mortality rates in infected poultries and chickens reaching as high as 100 percent (Chen et al., 2022, Fujimoto and Haga, 2024). While human infections are relatively rare, the H5N1 virus has shown a high fatality rate of approximately 50 percent in reported human cases globally, showcasing a pandemic potential in the future (Chen et al., 2022, Fujimoto and Haga, 2024, Musa et al., 2024). Economically, outbreaks have resulted in drastic losses, such as the estimated $ 3.3 billion impact of the 2014–2015 HPAI outbreak in the United States. Given the ongoing global circulation of Avian Influenza Viruses (AIVs) and their potential for evolution and transmission to humans, understanding the complex factors that result in these outbreaks is the key for developing effective prevention and control strategies (Morin et al., 2018, Fujimoto and Haga, 2024).

The transmission of HPAI is multi-factorial, meaning it involves a complex interplay of environmental, geographical, and human-related elements. In many studies, wild migratory birds are recognized as the primary natural cause and play a crucial role in the maintenance and intercontinental dispersal of AIVs, with their migration patterns contributing significantly to the seasonal and global distribution of the virus (Morin et al., 2018, Chen et al., 2022, Patyk et al., 2023, Fujimoto and Haga, 2024, Wang et al., 2025a). Proximity to wild waterfowl or shorebirds has been directly associated with increased odds of HPAI outbreaks in commercial farms (Patyk et al., 2023). Beyond wildlife, the proximity of poultry farms to one another is known to be a major factor, with the probability of outbreaks decreasing extensively as the distance between farms increases. This local area spread is often attributed to the movement of people and equipment, although wind-borne transmission is also increasingly investigated and recognized (Boender et al., 2007, Ssematimba et al., 2012, Ypma et al., 2012, Wells et al., 2017, Gkrinia et al., 2025).

Meteorological conditions are significant drivers of AIV outbreaks. Key climatic factors include:

• Temperature [°C]: research indicates varied relationships.Mainly, colder temperatures are more permissive for viral survival outside the host (Islam et al., 2023, Opata et al., 2025). Others report a protective effect of higher maximum temperature anomalies on outbreak occurrence. Highly pathogenic AIVs are known to be sensitive to higher temperatures and ultraviolet light, which can reduce their lifespan and ability to spread (Islam et al., 2023, Wang et al., 2025a).
• Humidity [%]: Relative Humidity (RH) is a critical factor, with low humidity often increasing the chance of influenza virus survival and transmission (Lowen et al., 2007, Aliabadi et al., 2011). Also, high specific humidity can act as a protective factor, as larger, heavier aerosol particles in humid air cause the virus to fall faster, limiting airborne spread (Peci et al., 2019, Chen et al., 2022). Absolute humidity has also been negatively associated with influenza virus infections (Peci et al., 2019).
• Wind speed [m s^−1^] and direction [°]: disease spread has been statistically correlated with wind direction at the time of infection. Certain low averaged wind speeds preceding an outbreak have been significantly associated with increased risk, potentially because higher wind speeds facilitate ventilation and dilution of virus concentrations in farms, thus acting as a protective factor (Ypma et al., 2012, Chen et al., 2022).
• Precipitation [mm h^−1^]: reduced precipitation has been observed to facilitate HPAI H5N1 occurrences in wild birds, likely because precipitation down-washes the virus from the atmosphere (Si et al., 2010).

Other geographical and environmental features also play a part in risk factors:

• Elevation [m]: lower elevations are frequently associated with the sub type H5N1 outbreaks (Si et al., 2010).
• Water bodies and infrastructure: proximity to water bodies is a recurring risk factor. Studies show that greater distance from water areas, roads, and railways can act as protective factors, limiting virus spread (Chen et al., 2022). Water bodies are known to attract wild birds, which can potentially transmit the virus. Highway networks have been identified as contributing to spread (Islam et al., 2023).
• Poultry density: high poultry density in a region or on a farm significantly increases the risk of outbreaks and supports virus maintenance (Musa et al., 2024, Gkrinia et al., 2025).

However, usually epidemiological models often rely on spatial cross sectional designs to evaluate these factors. While these models are effective at mapping general regional vulnerability (i.e., identifying where high risk zones are located based on static features like infrastructure, or baseline regional density), they frequently suffer from unmeasured spatial confounding. Consequently, they often fail to capture the transient, day to day triggers of an acute outbreak (i.e., identifying when a specific existing farm is in immediate danger of infection).

To the authors’ knowledge, there is a critical gap in the literature regarding the temporal modeling of HPAI outbreaks using self matched designs. While the primary environmental risk factors for AIV are studied, their complex non linear interactions and, moreover, how fluctuations in these weather patterns synchronize with spikes in local active infections, remain poorly understood. Recent work by Wang et al. (2025b) utilized machine learning (XGBoost) to identify temperature drivers in China; however, such approaches often rely on post-hoc interpretations rather than providing mathematically stable or explainable relationships. Furthermore, Huang et al. (2025) emphasized the need for integrating diverse environmental datasets to improve model generalizability without falling victim to spatial survivorship bias.

This study addresses these epidemiological gaps by deploying a time-stratified case-crossover design (Maclure, 1991). As outlined by Maclure and Mittleman (2000), this methodology is uniquely suited for evaluating intermittent exposures (e.g., meteorological fluctuations and dynamic proximity to active outbreaks) that create transient shifts in the risk of abrupt-onset events. By using each infected premise as its own control, this framework inherently eliminates time invariant spatial confounders. These may include elevation and static farm density that helps with the strict isolation of dynamic environmental and localized transmission triggers (Zhang et al., 2011, Zhang et al., 2023).

We implement this design through a conditional logistic regression framework (using the difference method), optimized with Ridge (L2) regularization. This approach quantifies the non-linear biological realities of viral survival, such as the saturation effect of infected neighbors and the atmospheric wash-out effect of precipitation. Furthermore, we analyze distinct regional transmission profiles across Canada by enforcing epidemiological Events Per Variable (EPV) [-] thresholds to prevent mathematical overfitting in smaller provincial datasets.

The paper is structured as follows. In Section “Materials and methods”, we detail our methodology, covering data sources, the case-crossover study design, and the mathematical implementation of the conditional difference method. In Section “Results”, we present the comparative performance of the optimized national model and the highly distinct environmental risk profiles revealed by the provincial-level stratification. In Section “Discussion”, we interpret these findings, analyzing the statistical evidence for wind-borne aerosol spread, the environmental shielding effect of precipitation, and the dominance of local proximity in dense agricultural zones. Finally, in Section “Conclusion, limitation, and future work”, we provide our conclusions and recommendations for future research and surveillance strategies.

## Materials and methods

### Data source

**Fig. 1:**
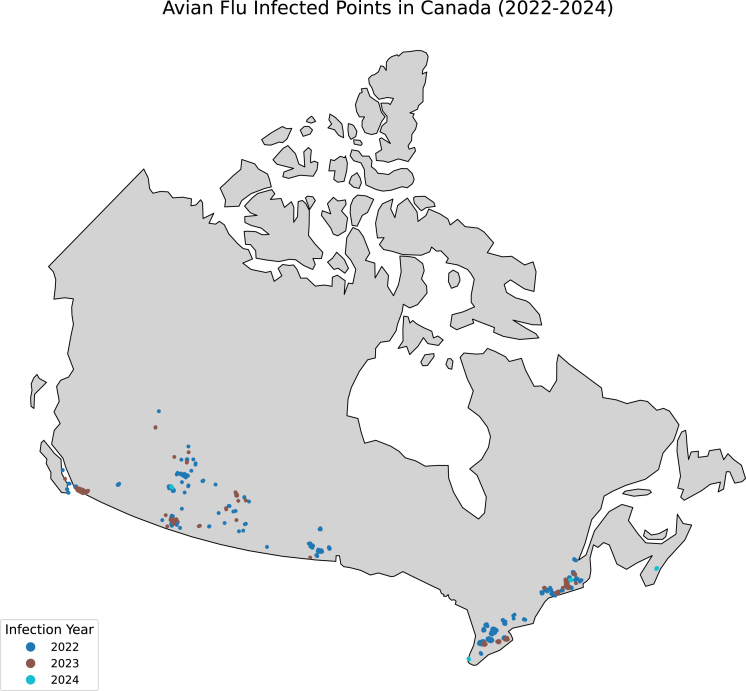
Outbreak locations across all of Canada from 2022 to 2024; map produced using geopandas (version 1.1.1) in Python.

The outbreak data in poultry facilities were obtained from a veterinary surveillance program. This dataset includes infection reports in individual farm facilities across Canada from 2022 to 2024. Fig. 1 shows the infection reports in a map across all locations of Canada from 2022 to 2024. In addition to the farm infection data, meteorological data, which includes air temperature (2 m above ground), dew point temperature (2 m above ground), wind velocity components (10 m above ground), and precipitation were collected using the ERA5 reanalysis dataset from the Copernicus Climate Change Service.^1^ The ERA5 dataset provides global estimates of atmospheric variables at a temporal resolution of 6 h and a horizontal resolution of approximately 31 km (Aliabadi and McLeod, 2023). These parameters were averaged for the 10-day period preceding infection reports, based on the findings from previous studies, given the incubation period of the virus. Static spatial variables, such as farm elevation, were ultimately excluded from this dataset, as their effects are mathematically factored out by the study design described in Section “Study design: Time-stratified case-crossover framework”.

### Dataset creation

The dataset was constructed by integrating farm-level and meteorological data using a Python script. This process involved several key steps. Farm data included infection times and geographical coordinates. This was then merged with meteorological data from the ERA5 dataset. Afterwards, functions were created to find the nearest meteorological data points according to the resolution of data (temperature [ °C], dew point temperature [ °C], wind velocity components [m s^−1^], and precipitation [mm h^−1^]) from the ERA5 dataset for each farm’s location and infection date. To ensure mathematical stability during subsequent polynomial feature engineering, absolute temperatures in Kelvin were converted to Celsius. Wind speed and direction were then calculated from the u [m s^−1^] (west to east) and v [m s^−1^] (south to north) components, and relative humidity was calculated from the temperature and dew point temperature.

Using feature engineering, several new features were created to better understand potential transmission pathways: (1) $NNF_{NW}$ [-]: this feature counts the number of farms within a 20-km radius, irrespective of wind direction; (2) $NNF_{I}$ [-]: this feature counts number of infected farms within a 20-km radius and within the past 10 days; (3) $NNF_{UWI}$ [-]: this feature counts the number of infected farms within a 20-km radius that are also upwind of the target farm and were infected within the past 10 days.

### Study design: Time-stratified case-crossover framework

Because the dataset strictly provided records of infected premises, a spatial cross-sectional design (comparing infected farms to a series of uninfected farms) was not feasible and would introduce spatial bias. Therefore, to evaluate the transient environmental and meteorological triggers of HPAI outbreaks, we utilized a time-stratified case-crossover design (Maclure, 1991).

As outlined by Maclure and Mittleman (2000), this methodology is specifically tailored for studying abrupt-onset events triggered by intermittent and transient exposures. By design, each infected premises serves as its own control. For each recorded outbreak (the ‘case’ period), a corresponding ‘control’ period was generated by randomly selecting a 10-day window for that exact same farm that fell entirely outside of its documented hazard and active infection periods. This self-matching framework acts as the ultimate form of statistical adjustment, effectively eliminating bias from time invariant measured and unmeasured spatial confounders, such as a farm’s exact geographic coordinates, local topography, and general static farm density (Zhang et al., 2011, Zhang et al., 2023).

### Model development and the difference method

To mathematically execute the case-crossover design within a machine learning classification framework, we employed the difference method, which replicates a conditional logistic regression. For every unique farm, the dynamic meteorological and proximity features of its control period were subtracted from the features of its outbreak period, creating a single vector of transient change:

(1) $$\Delta X=X_{case}-X_{control}$$

To ensure class balance during model training, the subtraction order was randomly reversed ($\Delta X=X_{control}-X_{case}$) for 50% of the paired samples to represent the negative class (Label = 0). By training the logistic regression directly on these ΔX vectors, the algorithm isolates the temporal shifts in environmental conditions (e.g., sudden changes in wind or the sudden appearance of an infected neighbor) that immediately precede an outbreak.

**Fig. 2:**
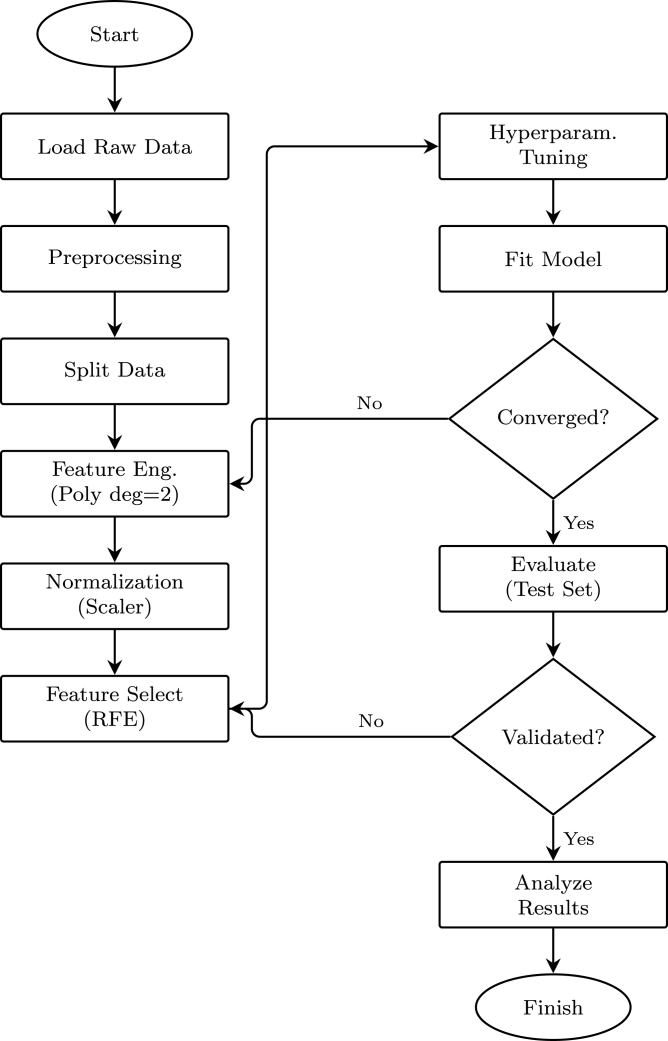
Methodology flowchart.

A multi-variable logistic regression model was employed for this analysis, chosen over alternatives such as Random Forests or Support Vector Machines for its high degree of interpretability (Huang et al., 2025). To prevent the aggressive elimination of correlated biological variables (e.g., temperature and its squared term, T and $T^{2}$), the model was penalized exclusively using Ridge (L2) regularization. Unlike Lasso (L1) regularization, which arbitrarily zeroes out correlated features, Ridge smoothly distributes weights, ensuring the resulting coefficients remain epidemiologically stable and interpretable. The conditional structure of the difference method imposes a zero intercept ($\beta_{0}=0$), so the linear predictor is a weighted sum of the transient feature differences alone. The detailed mathematical formulation, including the sigmoid link function, the maximum likelihood estimation procedure, and the Odds Ratio (OR) derivation, is provided in the supplementary material (Section S.2). All analyses were conducted in Python using scikit-learn; software environment details are also reported in Section S.2. Fig. 2 shows the methodology flowchart.

### Model validation and evaluation

Because the difference method collapses paired data into a single row per farm, a standard train–test split is completely immune to spatial data leakage. The dataset was partitioned into a training set (70% of the data) and a hold-out test set (30%) using province-level stratification.

Model development, hyperparameter tuning, and feature selection were performed exclusively on the training data using 5-fold cross-validation. Model performance on the unseen test set was assessed using the Area Under the Receiver Operating Characteristic Curve (ROC-AUC), balanced accuracy, precision, recall, F1-score, and the Brier score. Calibration curves were generated to assess the reliability of the predicted probabilities.

### Analytical approach: Three-step modeling progression

To identify the most robust risk factors, we evaluated three nested models of increasing complexity:

• **Baseline Linear Model (BLM):** A standard logistic regression using only the raw linear features. This established a performance floor and identified the strongest primary drivers.
• **Fully Engineered Model (FEM):** Expanded the BLM with all second-order polynomial and interaction terms (e.g., $T^{2}$, $S\times NNF_{I}$), producing over 40 candidate terms, of which 27 remained after removing constant and duplicate columns introduced by the polynomial expansion. This tested whether non-linear interactions improved explanatory power, though at the cost of multicollinearity and overfitting risk.
• **Enhanced Fully Engineered Model (FEM+):** Combined the engineered features of FEM with Recursive Feature Elimination (RFE) using a Ridge-penalized base estimator, selecting the most informative subset. Hyperparameters, including the optimal number of features to retain, were tuned jointly via cross-validation. FEM+ was selected as our final national model.

Implementation details, including the specific scikit-learn pipeline architecture and hyperparameter grids, are reported in the supplementary material (Section S.2).

### National and provincial-level analyses

The methodology outlined was applied to national and provincial-level analyses. For national-level analysis, we used all three models: BLM, FEM, and FEM+. To investigate potential regional variations in HPAI transmission, the complete model pipeline (FEM+) was applied to provincial subsets of the data. We hypothesized that the vast geographical and climatological differences across Canada would result in different localized risk profiles.

To ensure mathematical validity and prevent overfitting on smaller regional datasets, feature selection for the provincial models wasstrictly governed by the epidemiological Events Per Variable (EPV) [-] and the rule of 10. The maximum number of engineered features permitted for any provincial model was dynamically limited based on the total number of discordant outbreak pairs available in that province (e.g., a province with 35 pairs was restricted to a maximum of 3 features). Provinces possessing fewer than 15 discordant pairs (e.g., Nova Scotia) were excluded from independent regional analysis due to sample size limitations. For each qualifying province, the GridSearchCV pipeline was independently re-fitted, allowing the algorithm to identify the most significant regional risk factors.

## Results

The objective of this analysis was to isolate and quantify the transient environmental and localized transmission triggers of Avian Influenza (AI) outbreaks across Canada.

### Descriptive statistics and temporal baselines

Prior to machine learning feature selection, a descriptive analysis of the raw dynamic variables was conducted to establish the baseline differences between control dates (safe periods) and outbreak dates (infection periods) for the paired premises. Table 1 highlights a stark, observable shift in local proximity metrics: the mean number of actively infected neighboring farms within a 20-km radius ($NNF_{I}$ [-]) jumped from 17.13 on control dates to 237.23 on outbreak dates. Similarly, the mean number of upwind infected neighbors ($NNF_{UWI}$ [-]) surged from 2.05 to 31.22, showing a potential raw evidence of clustered or wind-facilitated spread.

**Fig. 3:**
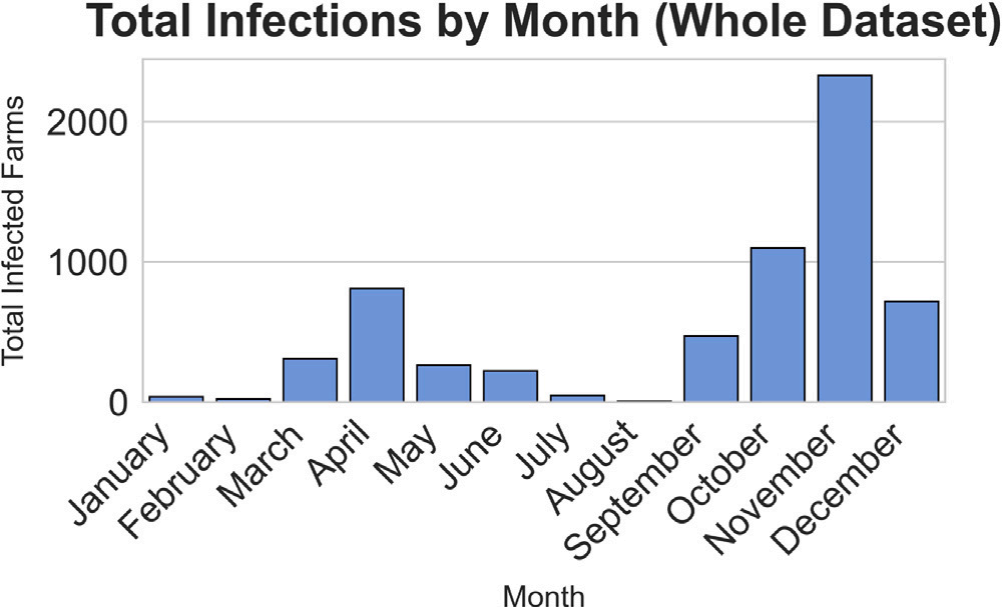
Outbreaks by month across Canada.

Additionally, raw outbreak frequencies showed a seasonal distribution, peaking in the spring and fall months, consistent with known migratory bird patterns and semi cool weather viral survival limits. This is illustrated in Fig. 3, Fig. 4.

**Table 1:** Descriptive statistics (mean and standard deviation) of key dynamic environmental and transmission variables across paired Control (No. Infected) and Outbreak (Infected) dates.

Variable	Control dates (P = 0)	Outbreak dates (P = 1)
Temperature [°C]	9.32±8.83	6.04±6.05
Relative Humidity [%]	74.16±13.34	76.14±12.55
Wind Speed [m s^−1^]	1.92±1.38	2.29±1.42
Precipitation [mm h^−1^]	0.00±0.00	0.00±0.00
$NNF_{I}$ (Number of Infected Neighbors) [-]	17.13±71.18	237.23±221.64
$NNF_{UWI}$ (Number of Upwind Infected Neighbors) [-]	2.05±11.99	31.22±55.87

Fig. 4Outbreaks per month for highly impacted provinces.Fig. 4

**(a):**
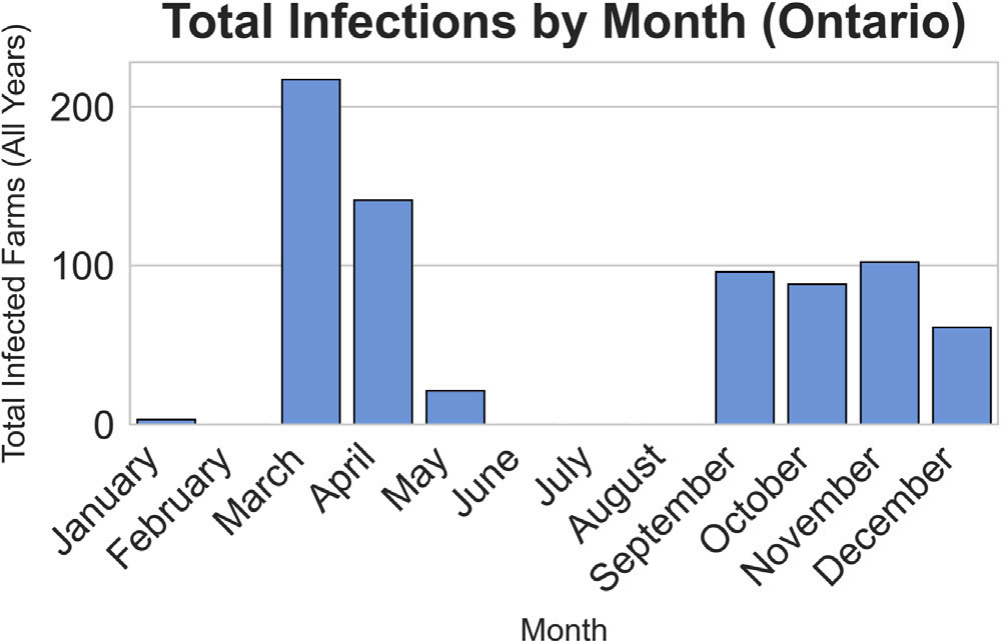
Ontario.

**(b):**
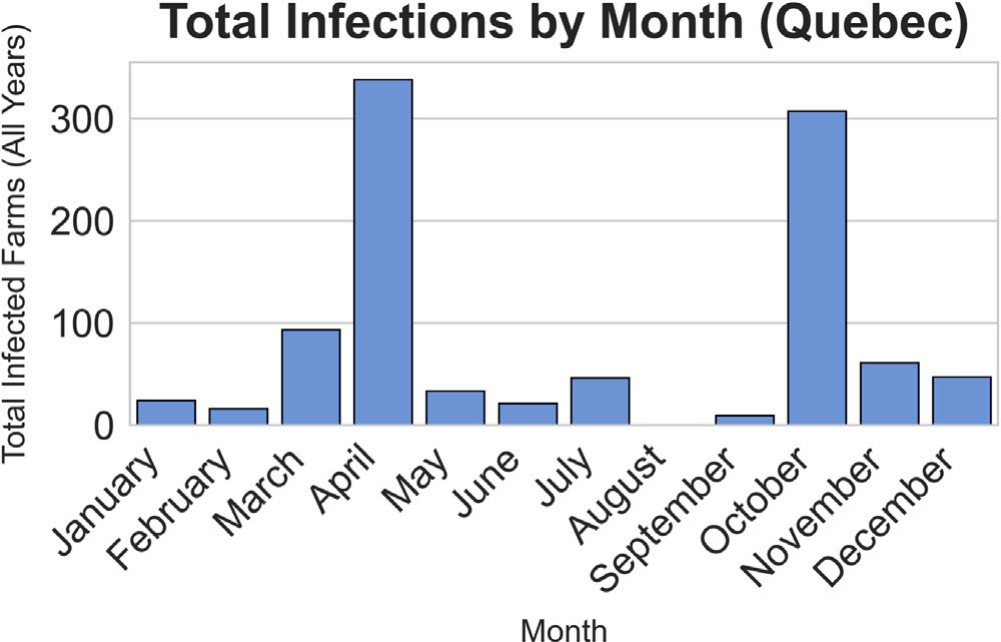
Quebec.

**(c):**
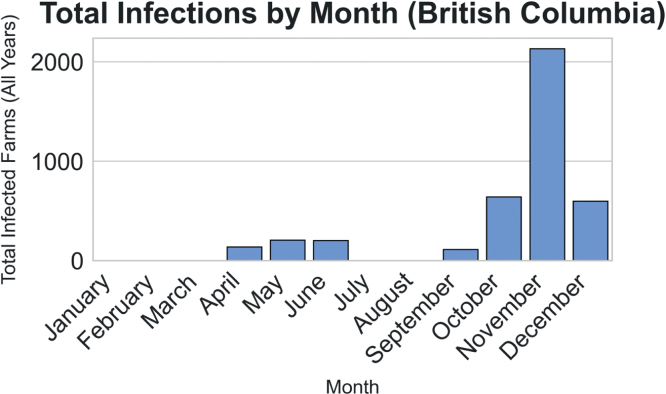
British Columbia.

### National model progression and validation

As mentioned, to identify the most robust risk factors, we evaluated three iterations of the conditional logistic regression framework: the Baseline Linear Model (BLM), the Fully Engineered Model (FEM), and the Enhanced Fully Engineered Model (FEM+).

The BLM, restricted only to raw linear variables, established a baseline understanding but failed to capture the synergistic effects of weather and local farm density. The FEM incorporated all second-order polynomials and interactions (over 40 candidate terms, reduced to 27 after dropping degenerate columns), which increased explanatory power but suffered from severe multicollinearity and a high risk of overfitting. Finally, the optimized FEM+ model, utilizing Ridge (L2) Recursive Feature Elimination, successfully stripped away statistical noise to isolate a more stable set of 10 features.

**Fig. 5:**
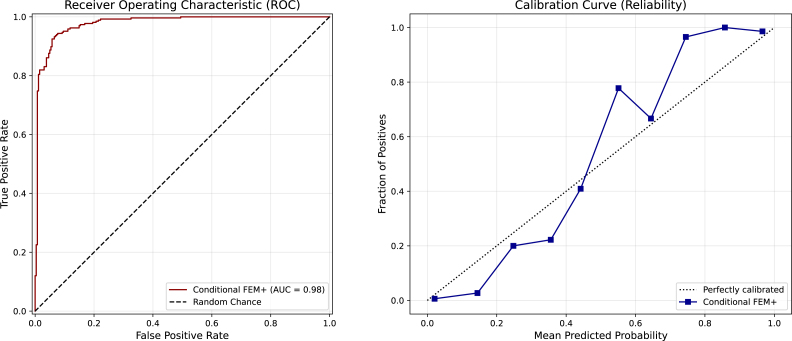
National FEM+ Model Validation. (Left) The Receiver Operating Characteristic (ROC) curve demonstrating excellent discriminative ability (AUC = 0.978). (Right) The Calibration Curve proving predicted probabilities tightly align with true outbreak frequencies, resulting in a highly reliable Brier Score (0.060).

On the hold-out test set, the FEM+ model achieved an ROC-AUC of 0.978, a balanced accuracy of 0.932, and a Brier score of 0.060, indicating both strong discriminative ability and well-calibrated probability outputs (Fig. 5). The model demonstrated stability under 5-fold cross-validation, achieving a mean validation ROC-AUC of 0.969 ± 0.011. Additional validation diagnostics, including the McFadden’s Pseudo $R^{2}$, AIC-based parsimony comparison between FEM and FEM+, and Variance Inflation Factor (VIF) analysis, confirming the absence of structural multicollinearity, are reported in the supplementary material (Section S.1).

### National analysis

**Fig. 6:**
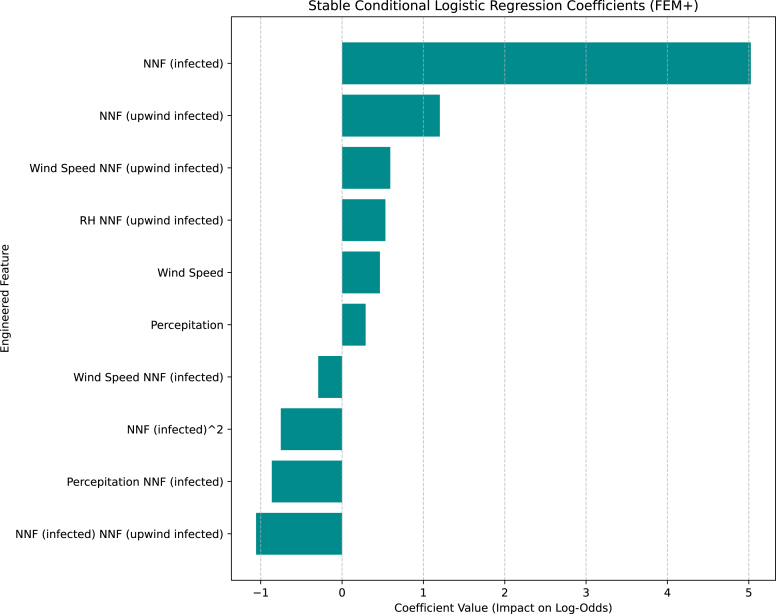
Coefficient plot for the stable Conditional FEM+ model, showcasing the primary drivers of HPAI infection.

The 10 engineered features selected by the national FEM+ model show the physical and biological mechanics of HPAI transmission. To facilitate interpretation, the resulting log-odds coefficients (β) were converted into Odds Ratios ($OR=e^{\beta }$ [-]), which quantify the multiplicative change in infection risk. These are presented in Table 2 and visualized in Fig. 6.

The analysis of these interactions hypothesizes four epidemiological mechanisms driving outbreaks across Canada:

**Table 2:** Final optimal features, log-odds coefficients (β), and Odds Ratios (OR) for the national FEM+ model.

Engineered feature	Coefficient (β)	Odds Ratio (OR)
$NNF_{I}$ [-]	＋5.0249	152.16
$NNF_{UWI}$ [-]	＋1.2013	3.32
$S\;\times \;NNF_{UWI}$ [m s^−1^]	＋0.5921	1.81
$RH\;\times \;NNF_{UWI}$ [%]	＋0.5325	1.70
S (Wind Speed) [m s^−1^]	＋0.4653	1.59
P (Precipitation) [mm h^−1^]	＋0.2896	1.34
$S\;\times \;NNF_{I}$ [m s^−1^]	−0.2931	0.75
$NNF_{I}^{2}$ [-]	−0.7530	0.47
$P\;\times \;NNF_{I}$ [mm h^−1^]	−0.8636	0.42
$NNF_{I}\;\times \;NNF_{UWI}$ [-]	−1.0564	0.35

• The Proximity Baseline: The most important driver of an outbreak is the sudden appearance of an actively infected neighboring farm. This shows that while broad environmental factors are permissive, active local clusters are the acute triggers.
• Wind-Borne Spread: The model provides a statistical evidence of wind-mediated viral transport. The presence of an upwind infected neighbor ($NNF_{UWI}$ [-]) multiplies the baseline risk by an additional factor of 3.32.
• Precipitation: The model identified a protective interaction between precipitation and local outbreak density ($P\times NNF_{I}$ [mm h^−1^], OR = 0.42). Because the Odds Ratio (OR) [-] is below 1.0, this indicates that during a local cluster outbreak, concurrent precipitation events reduce the odds of farm to farm transmission.
• Saturation: The negative quadratic term for infected neighbors ($NNF_{I}^{2}$ [-], OR = 0.47) potentially models an epidemiological saturation curve.

### Provincial level analysis

To test the hypothesis that if the HPAI transmission mechanics vary geographically, the FEM+ pipeline was independently applied to the discordant pairs of individual provinces. The results, summarized in Table 3, with the corresponding provincial discrimination shown in Fig. 8, reveal two highly distinct geographical transmission profiles.

**Table 3:** Summary of provincial FEM+ conditional models. Maximum allowed features were strictly capped by the Rule of 10. Explanatory ROC-AUC evaluates the model’s ability to separate safe days from outbreak days within the regional paired sample.

Province	Outbreak pairs	Max EPV limit	ROC-AUC	Selected key drivers
Ontario (ON)	567	56	0.9951	$NNF_{I}$, $NNF_{UWI}$, S, P, $NNF_{UWI}^{2}$
British Columbia (BC)	582	58	0.9642	$NNF_{I}$, $NNF_{UWI}$, $NNF_{I}^{2}$, $NNF_{I}\;\times \;NNF_{UWI}$
Quebec (QC)	295	29	0.9869	$NNF_{I}$, $NNF_{UWI}$, $RH\;\times \;NNF_{UWI}$
Alberta (AB)	129	12	0.9695	$NNF_{I}$, $S\;\times \;NNF_{I}$, S
Manitoba (MB)	138	13	1.0000	$NNF_{I}$, $NNF_{UWI}$, T×P
Saskatchewan (SK)	35	3	0.9933	$NNF_{I}$, $T^{2}$, $RH^{2}$

**Fig. 7:**
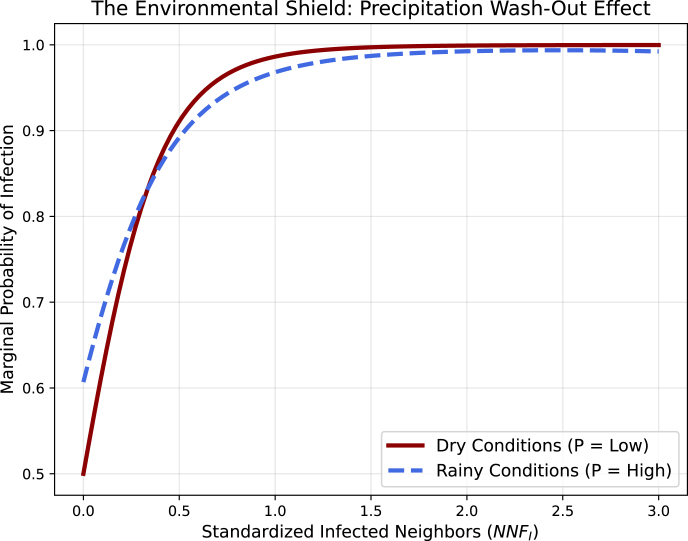
Marginal effect interaction plot demonstrating the Environmental Shield. Under dry conditions (red), the probability of infection surges rapidly with the appearance of infected neighbors. Concurrent precipitation (blue) suppresses this curve, cutting the transmission odds by physically washing viral aerosols from the local atmosphere.

**Fig. 8:**
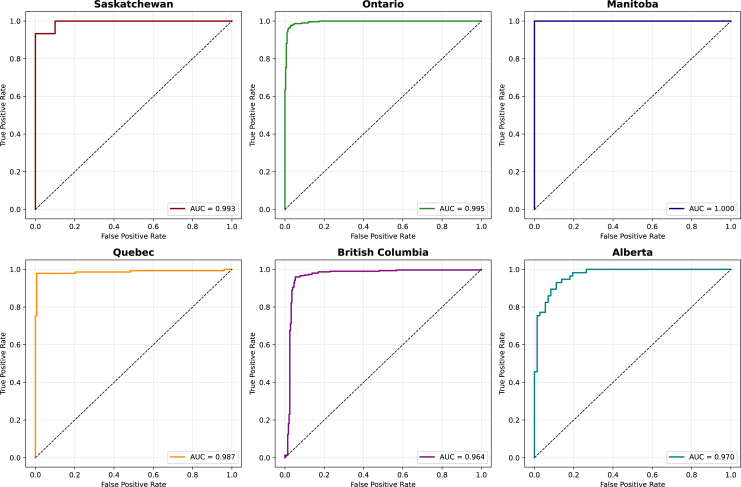
ROC curves for the provincial models, demonstrating a valid predictive stability despite geographical and climatic variations. Only provinces with sufficient paired observations satisfying the EPV threshold were independently modeled.

## Discussion

Before discussing the epidemiological implications of our findings, we wish to emphasize that all interpretations in this section describe statistical associations identified by the conditional logistic regression model. While these associations are consistent with biologically plausible mechanisms — including wind-mediated aerosol transport, precipitation-driven viral wash-out, and density driven local transmission, our observational case-crossover design does not provide direct experimental evidence for these mechanisms. The mechanistic interpretations offered throughout this section should therefore be regarded as hypotheses generated by the data.

The results of this study, specifically the FEM+ as applied within the conditional difference method, provide insights into the complex transmission dynamics of Highly Pathogenic Avian Influenza (HPAI). By utilizing a time-stratified case-crossover design, we successfully eliminated time-invariant spatial confounding, allowing for the strict isolation of the transient, day to day environmental and localized triggers of an outbreak. Below, we discuss the epidemiological implications of the national and provincial risk profiles.

### National risk profile

Unlike traditional spatial models, this conditional approach reveals that the immediate trigger for an outbreak is rarely an isolated meteorological event, but rather the synergistic interaction between specific weather conditions and active local clusters.

The model identified the sudden appearance of an actively infected neighbor ($NNF_{I}$ [-]) as the strongest statistical predictor. However, the model’s non-linear engineering showed a critical biological reality: The associated risk does not scale infinitely in our model. The negative quadratic term ($NNF_{I}^{2}$ [-], OR = 0.47) mathematically describes is consistent with an epidemiological saturation pattern. This indicates that while proximity to an infected farm is the dominant risk factor, this risk plateaus at high densities. This is likely due to two major reasons. First, is the biological saturation effect: if a farm is already surrounded by a high density of infected premises, its environmental exposure is already maximized; adding another infected neighbor marginally changes the baseline viral load. Second, is human intervention: in areas with a high density of outbreaks, biosecurity measures enforced by both farmers and the relevant authorities become exceptionally strict. This includes rapid movement controls, aggressive sanitation, and preemptive bird culling (depopulation), which actively suppresses further transmission.

#### Statistical patterns consistent with wind-mediated transmission

A notable finding of this study is a statistical pattern consistent with wind-mediated viral transport, though the observational design precludes direct causal inference. The model isolated the upwind infected neighbor ($NNF_{UWI}$ [-]) as a distinct and massive risk amplifier. In other words, this upwind risk is supercharged by two specific meteorological engines: wind speed ($S\times NNF_{UWI}$ [m s^−1^], OR = 1.81) and relative humidity ($RH\times NNF_{UWI}$ [%], OR = 1.70). High wind speeds physically facilitate the atmospheric dispersion of plumes from the upwind premises, while elevated relative humidity prevents the rapid desiccation and decay of the aerosolized viral particles in transit (Lowen et al., 2007, Peci et al., 2019). This triad of features is consistent with short to medium range airborne transmission as a candidate risk during cluster outbreaks, pending experimental validation. This suggests that future biosecurity guidelines may benefit from considering the strategic orientation of barn intake and exhaust fans.

#### The environmental shield

Other than wind-borne transmission, the model identified a powerful protective interaction between local outbreak density and precipitation ($P\times NNF_{I}$ [mm h^−1^], OR = 0.42). Because the Odds Ratio (OR) [-] is substantially below 1.0, the model identifies a statistical association whereby concurrent precipitation is associated with approximately 58% lower odds of co-occurring transmission events. This association is consistent with atmospheric wash out as a candidate mechanism, whereby heavy rainfall may physically scrub dust, dander, and viral particles from the air, effectively neutralizing the airborne transmission corridor between adjacent farms (Si et al., 2010). This interaction is illustrated in Fig. 7 under dry conditions the probability of infection rises sharply as infected neighbors appear, whereas concurrent precipitation flattens that curve.

#### Temperature’s role

Notably, raw temperature (T [ °C]) was eliminated as a primary linear feature by the Ridge RFE algorithm. While this appears to contradict previous spatial studies, it actually highlights the strength of the case-crossover design. Temperature acts as a seasonal permissive factor (setting the broad environmental conditions for wild bird migration and viral stability over months). However, on a day to day basis for a specific farm, the ambient temperature difference (ΔT [ °C]) between a safe day and an infected day is negligible compared to the sudden transient triggers of a neighbor becoming infected.

### Provincial risk profiles

This study provides a statistical confirmation that HPAI transmission is not a uniform, national process, but a series of distinct regional phenomena. The provincial-level analysis revealed two distinct transmission archetypes across Canada. This finding has critical implications for surveillance, indicating that biosecurity strategies must be tailored to regional agricultural geography. We note that the perfect explanatory ROC-AUC for Manitoba (Table 3) reflects in-sample separation of discordant pairs within a small regional subset; it is reported as a measure of within sample explanatory fit and should not be read as out of the sample predictive accuracy.

#### Profile A

In highly consolidated poultry-producing regions, such as Southern Ontario, the Fraser Valley in British Columbia, and the St. Lawrence River Valley in Quebec, the models were almost entirely governed by proximity ($NNF_{I}$ [-]) and wind-direction ($NNF_{UWI}$ [-]) metrics, discarding most ambient meteorological variables. We shall define this as Density Override effect. In these dense agricultural zones, the baseline risk of being situated near an active cluster is so extreme that localized transmission vectors, such as the movement of shared feed trucks, personnel, equipment, or direct short-range wind dispersion, supersede the influence of general weather conditions. For example, in Quebec and Ontario, the overwhelming dominance of $NNF_{I}$ [-] and $NNF_{UWI}$ [-] suggests that the congestion and alignment of farms matters far more than transient changes in temperature or humidity. The prominence of upwind metrics ($NNF_{UWI}$ [-]) in Quebec may also suggest that aerosol plumes are the primary vector, likely channeled by the topography of the St. Lawrence River Valley. Consequently, outbreak management in these provinces must prioritize extreme physical quarantine and immediate logistical lockdown of local corridors.

#### Profile B

In contrast, despite low number of pairs, Saskatchewan (and somewhat Manitoba for T×P [°Cmm h^−1^]) were the only provinces where the model retained ambient meteorological features ($T^{2}$ [${}^{\circ}C^{2}$], $RH^{2}$ [%${}^{2}$], P [mm h^−1^]) alongside proximity. This reflects the unique agricultural geography of the open Prairies. Because farms in Saskatchewan are separated by vast geographic distances, the dense, farm to farm cluster outbreaks seen in Ontario or BC, are inherently restricted. In this dispersed landscape, the virus relies heavily on specific, highly permissive ambient weather conditions to remain viable over long distances (whether transported by wild birds or human vectors). This implies that surveillance in the more isolated Prairie regions must be highly dynamic, with biosecurity protocols escalating specifically during weather events that favor prolonged viral stability in the environment. To further solidify this finding, a real world, precise analysis in the prairies might be needed, preferably, with a higher number of data points.

### Practical implications for poultry biosecurity

While our analysis is observational and the mechanistic interpretations described above remain hypotheses requiring field validation, the statistical patterns we identified are sufficiently consistent across provinces and analytical approaches to inform several practical considerations for biosecurity practice. We translate our findings into region-specific operational recommendations below, framed as hypotheses for producers, veterinarians, and policymakers to evaluate against their own operational contexts.

#### Recommendations for density-override regions (Ontario, British Columbia, Quebec)

In regions where our analysis identified $NNF_{I}$ and $NNF_{UWI}$ as the dominant statistical drivers, we suggest:

• Proximity-triggered biosecurity escalation: The dominance of$NNF_{I}$ (OR = 152) in our model suggests that the declaration of an infected premise within approximately 20 km should prompt immediate biosecurity escalation at neighboring farms, rather than waiting for direct epidemiological links. This is consistent with current control-zone policy but supports tightening of the radius and shortening of the response window. It is also worth mentioning that the effective radius could be altered according to field studies.
• Ventilation operation: The statistical pattern consistent withwind-mediated transmission ($S\times NNF_{UWI}$, OR = 1.81) suggests that during active regional outbreaks, producers may wish to change ventilation rate as well as the orientation and location of barn intake and exhaust systems, relative to prevailing winds and to neighboring premises. We must emphasize that this is a hypothesis driven recommendation; experimental validation through air trajectory studies is required before this becomes a formal biosecurity standard. If the hypothesis is validated with high statistical significance via field studies, new designs for ventilation systems can be developed.
• Operational risk windows: The protective association between precipitation and transmission ($P\times NNF_{I}$, OR = 0.42) suggests that dry, low-humidity periods during active regional outbreaks may represent elevated short-range transmission risk windows. Activities involving inter farm movement (feed delivery, technical visits, equipment transfers) may be scheduled in a way to avoid such windows where operationally feasible. This protective action is genuinely actionable but operationally hard; however, it could be eased with installation of measurement equipment after field cost–benefit studies.

#### Recommendations for meteorologically-dominant regions (Saskatchewan, Manitoba)

In dispersed Prairie regions where our analysis retained ambient meteorological features ($T^{2}$, $RH^{2}$, P, T×P) alongside proximity, we suggest:

• Weather triggered surveillance escalation: The retention of ambient weather features in the Prairie models suggests that surveillance intensity could be dynamically modulated based on regional weather conditions, with escalation during meteorological windows favoring prolonged viral viability (lower or optimal temperatures, sustained high humidity, prolonged dry spells).
• Migratory season vigilance: Given the seasonal outbreak distribution observed in our data and also the limited density driven transmission in these regions, biosecurity protocols may benefit from formal escalation during spring and fall migratory windows, with relaxation during low risk summer and mid-winter periods. This part will possibly be further investigated in our future studies.

#### Recommendations for federal and provincial policymakers

The two distinct transmission archetypes we identified have implications beyond individual farm practice:

• Regional biosecurity policy: Our analysis provides quantitative support for moving away from uniform national biosecurity standards toward regionally tailored frameworks. The factors that statistically dominate in dense agricultural zones differ from those in dispersed regions, and policy frameworks that fail to acknowledge this distinction risk being either over-restrictive in regions with low density or under-protective in the ones with high density.
• Investment in directional surveillance data: The statistical importance of upwind proximity ($NNF_{UWI}$) in our model highlights the operational value of integrating real time wind and meteorological data into outbreak surveillance dashboards. For example, wind anemometers can be installed at each farm. Such integration would allow control authorities to prioritize inspection of downwind premises during active outbreaks.
• Quadratic saturation as evidence of effective containment: The negative quadratic term on $NNF_{I}$ ($NNF_{I}^{2}$, OR = 0.47) is consistent with effective biosecurity intervention at high outbreak densities. This statistical signature suggests that current containment protocols, while imperfect, are exerting measurable downward pressure on transmission in cluster zones and perhaps supporting continued investment in rapid response capacity.

We emphasize that all recommendations above are derived from associational statistical patterns and should be regarded as data informed hypotheses for further evaluation, not as proven interventions. Field validation, cost–benefit analysis, and coordination with existing authorities would be required before any of these become formal biosecurity standards.

## Conclusion, limitation, and future work

### Conclusion

This study demonstrated that the acute transmission of Highly Pathogenic Avian Influenza (HPAI) in Canadian poultry farms is driven by complex, non-linear, and localized transient risk factors. While prior studies generally characterize temperature and humidity as primary linear drivers of viral persistence, our conditional analysis redefines the role of ambient weather. We conclude that temperature acts primarily as a broad, seasonal permissive factor. The actual daily trigger of an outbreak is mostly dominated by local farm proximity. Furthermore, our study contributes to the ongoing investigation of wind-borne transmission by identifying statistical patterns consistent with wind-mediated spread as a potential accelerator. This could be more solidified with further experimental or air trajectory studies from farm to farm. The presence of an upwind infected neighbor ($NNF_{UWI}$ [-]), particularly when high wind speeds and relative humidity, massively amplified transmission odds. In addition, we identified a novel environmental shield effect, quantifying that concurrent precipitation events may reduce the odds of farm-to-farm transmission, likely by scrubbing infectious aerosols from the local atmosphere.

Crucially, the central contribution of this research is the statistical confirmation that this national-level framework is insufficient on its own, as transmission mechanics are highly dependent on regional agricultural geography. We discovered two distinct provincial transmission archetypes. In highly consolidated poultry regions (Ontario, British Columbia, and Quebec), a density override effect occurs. However, in the open and highly dispersed geography (Saskatchewan and Manitoba), ambient meteorological factors remain critical drivers, as the virus relies heavily on specific weather windows to remain viable over vast distances.

In conclusion, this work provides a rigorous mathematical framework for Canadian policymakers to move beyond a generalized national response. Biosecurity interventions must be regionally tailored: focusing on extreme logistical lockdown and aerosol mitigation in dense provinces, while deploying dynamic and weather responsive surveillance in dispersed regions.

### Limitation

This study acknowledges several important limitations that should be considered when interpreting our findings. First of which is spatial resolution of meteorological data. The ERA5 reanalysis dataset possesses a spatial resolution of approximately 31 km. While this provides a continuous and reliable atmospheric baseline and avoids the severe missing data issues associated with sparse rural weather stations, this resolution is coarser than the typical farm-to-farm distances within consolidated poultry-producing regions. As a consequence, ERA5 cannot resolve microclimate variations that may matter for short-range aerosol transport, including thermal stratification near barn structures, local turbulence around windbreaks, or topographic channeling of airflow in valleys such as the Fraser or St. Lawrence. This limitation likely attenuates the magnitude of our wind-related associations rather than introducing systematic bias; nonetheless, the statistical patterns we identified should be regarded as conservative estimates of the underlying effects. Higher-resolution meteorological data, such as outputs from regional numerical weather prediction models or in-situ measurements from farm-mounted sensors, would substantially improve the resolution of future analyses.

Moreover, The dataset records the date of outbreak reporting or confirmation, rather than the exact biological moment of viral introduction. While we implemented a 10-day averaged lag window to account for the typical HPAI incubation period and passive surveillance delay, this approach assumes a relatively uniform reporting lag across all premises and provinces. In practice, reporting latency varies with farm size, biosecurity culture, regional veterinary capacity, and seasonal workload. This temporal smearing may obscure short-lag triggers and shift the timing of meteorological associations away from their true causal windows. Future research utilizing active genomic surveillance or daily serological testing could substantially refine these temporal triggers and distinguish true incubation dynamics from administrative reporting delays.

The time-stratified case-crossover framework is uniquely powerful for isolating transient triggers while controlling for time-invariant spatial confounders, but it carries inherent inferential limitations. First, our design inherently analyzes only those premises that eventually experienced an outbreak; we cannot identify factors that prevent viral introduction entirely for the farms that did not get infected. Future research incorporating spatial cohorts of strictly uninfected farms could help characterize baseline structural or geographical protective factors. Second, like all observational designs, case-crossover analyses identify statistical associations rather than proven causal mechanisms. The mechanistic interpretations offered in our Section “Discussion” should therefore be regarded as biologically plausible hypotheses generated by the data, requiring corroboration through controlled experimental studies or directly-measured air trajectory analyses.

Other than that, our dataset combines outbreaks across diverse poultry operation types, including broiler, layer, breeder, and small-flock premises. Different production types may exhibit substantially different biosecurity profiles, building designs, and exposure pathways, and the risk factors that dominate may differ between them. Production-type information is partially redacted in publicly available premises records to protect farm anonymity, which constrains stratified analysis. Future work with access to production-type metadata, conducted in collaboration with industry associations and veterinary authorities, could meaningfully refine these archetypes.

To conclude, several of the limitations described above ultimately reduce to a single underlying issue: the depth and granularity of analysis possible in HPAI epidemiology is fundamentally bounded by the access researchers have to detailed surveillance data and the operational context in which outbreaks occur. Substantive progress on mechanistic validation, regional refinement, and translation of statistical findings into operational protocols will require sustained collaboration between academic researchers, federal and provincial veterinary authorities, producer organizations, and individual poultry operators. We encourage data custodians and industry stakeholders to consider structured collaboration with research teams as a long-term investment in the resilience of the Canadian poultry sector. Such collaboration may include access to production type and biosecurity protocol metadata, participation in meteorological monitoring studies mounted in farms, and engagement in field validation of the data-informed hypotheses presented in this work. We welcome inquiries and partnerships from any organization seeking to extend or operationalize the findings reported here.

### Future work

Future work should involve the integration of active and daily genomic surveillance to precisely pinpoint the temporal latency of viral introduction. Expanding geographical regions of influence, coupling these machine learning models with advanced air trajectory and plume dispersion modeling, and analyzing shifting wild bird migration patterns under future climate conditions will be critical next steps in anticipating and mitigating the evolving threat of HPAI.

## CRediT authorship contribution statement

**Iman Sekhavati:** Writing – review & editing, Writing – original draft, Software, Methodology, Investigation, Data curation, Conceptualization. **Rozita Dara:** Writing – review & editing, Supervision. **Shayan Sharif:** Writing – review & editing, Supervision, Funding acquisition. **Amir A. Aliabadi:** Writing – review & editing, Writing – original draft, Visualization, Validation, Supervision, Software, Methodology, Investigation, Funding acquisition, Formal analysis, Data curation.

## Declaration of Generative AI and AI-assisted technologies in the writing process

During the preparation of this work, the author(s) used GPT-5.1 for typesetting equations and tables in LaTeX. The author(s) reviewed and edited the output as needed and take full responsibility for the content of the published article.

## Declaration of competing interest

The authors declare that they have no known competing financial interests or personal relationships that could have appeared to influence the work reported in this paper.

## Data Availability

The Atmospheric Innovations Research (AIR) Laboratory at the University of Guelph may provide the model source code and data, subject to approval from the data owners. For access, contact Amir A. Aliabadi ( aaliabad@uoguelph.ca), visit http://www.aaa-scientists.com/, or visit https://github.com/AmirAAliabadi.

## Supplementary data

MMC S1

## Footnotes

1 https://cds.climate.copernicus.eu/datasets/reanalysis-era5-single-levels?tab=overview.
