# Supplementary material for: Evaluating transient risk factors for avian influenza outbreaks in Canada using case-crossover study and machine learning
Source: Poult Sci. 2026 Jul 7;105(11):107364. doi: 10.1016/j.psj.2026.107364 (PMC13452994; doi:10.1016/j.psj.2026.107364)
Supplement: Supplementary file 1 [file mmc1.pdf]

Evaluating Transient Risk Factors for Avian Influenza Outbreaks in Canada Using Case-Crossover Study and Machine Learning (Supplementary Material)

Iman Sekhavati[a], Rozita Dara[b], Shayan Sharif[c], Amir A. Aliabadi[a,*]

[a] *Department of Mechanical Engineering, College of Engineering, University of Guelph, Guelph, Ontario, Canada*
[b] *Department of Computer Science, College of Computational, Mathematical and Physical Sciences, University of Guelph, Guelph, Ontario, Canada*
[c] *Department of Pathobiology, Ontario Veterinary College, Guelph, Ontario, Canada*

OVERVIEW

This supplementary document contains details, extended validation diagnostics, and mathematical formulations that support the analyses reported in the main manuscript. All section, figure, table, and equation labels in this file are prefixed with "S" to distinguish them from main-text references.

S.1. EXTENDED VALIDATION DIAGNOSTICS FOR THE NATIONAL FEM+ MODEL

This section reports additional validation diagnostics referenced in Section 3.2 of the main manuscript. These diagnostics support the parsimony, stability, and interpretability of the national FEM+ model and address standard concerns regarding generalization, goodness of fit, model selection, and multicollinearity in regularized polynomial regression.

S.1.1. Cross-Validation Stability

During 5-fold cross-validation on the training set, the FEM+ model achieved a mean validation ROC-AUC of 0.969 ± 0.011. The low variance across folds confirms that the model's discriminative performance is consistent across data partitions and does not depend on any particular subset of paired observations.

S.1.2. McFadden's Pseudo R^2

The model achieved a McFadden's Pseudo R^2 of 0.6326, computed as

$$R^2_{\text{McFadden}} = 1 - \frac{\ell_{\text{model}}}{\ell_{\text{null}}}, \tag{S.1}$$

where ℓ_{model} and ℓ_{null} denote the log-likelihoods of the fitted model and the null (intercept-only) model, respectively. For epidemiological logistic regression, values between 0.2 and 0.4 typically indicate an excellent fit (Hosmer Jr. et al., 2013); the observed value of 0.63 reflects strong alignment between the model and the paired transient data.

---

*Corresponding author

Email addresses: `isekhava@uoguelph.ca` (Iman Sekhavati), `drozita@uoguelph.ca` (Rozita Dara), `shayan@uoguelph.ca` (Shayan Sharif), `aaliabad@uoguelph.ca, +1(519)824-4120 x.54862, RICH 2515, 50 Stone Rd. East, University of Guelph, Guelph, ON, N1G 2W1, Canada` (Amir A. Aliabadi)

URL: `www.aaa-scientists.com` (Amir A. Aliabadi)

S.1.3. Parsimony: AIC Comparison Between FEM and FEM+

For completeness we report the Akaike Information Criterion (AIC) for both models: 892.65 for the FEM (27 features) and 914.43 for the FEM+ (10 features). We note that AIC, which rewards in-sample likelihood, nominally favors the larger FEM. We nonetheless selected FEM+ on the basis of generalization and interpretability rather than in-sample fit: removing 17 collinear, largely redundant terms substantially reduces overfitting risk and multicollinearity (see the VIF analysis in Section S.1.4) and yields an epidemiologically interpretable feature set, at only a modest cost in penalized in-sample likelihood.

S.1.4. Multicollinearity: Variance Inflation Factor

Although engineered polynomial features inherently possess some structural multicollinearity, a Variance Inflation Factor (VIF) analysis confirmed that the maximum VIF among the final 10 selected features was strictly bounded at 2.12, well below the common threshold of 5.0 indicating problematic multicollinearity. This confirms that the exclusive use of Ridge ($L2$) regularization successfully penalized structural inflation and ensured the log-odds coefficients in the results remain mathematically stable and epidemiologically interpretable.

S.2. MATHEMATICAL FORMULATION AND IMPLEMENTATION DETAILS

This section provides the complete mathematical formulation and implementation details referenced in Section 2.4 of the main manuscript.

S.2.1. Logistic Regression and the Sigmoid Function

Logistic regression predicts a binary outcome based on a set of independent variables using a logistic (sigmoid) link function:

$$p(x) = \frac{1}{1 + e^{-z}}, \tag{S.2}$$

where $p(x)$ is the probability of the outcome being 1 (infection) and z is a linear combination of the input features and their coefficients.

In the context of the conditional difference method employed in this study, the intercept (β_0) is strictly constrained to zero. The linear predictor is therefore composed exclusively of the transient feature differences (ΔX_i) and their corresponding coefficients:

$$z = \beta_1 \Delta X_1 + \beta_2 \Delta X_2 + \cdots + \beta_n \Delta X_n. \tag{S.3}$$

Features were standardized prior to any polynomial engineering to prevent structural multicollinearity. A positive coefficient ($\beta_i > 0$) indicates that an increase in the feature value is associated with an increased probability of infection. The magnitude of β_i reveals the relative impact of the feature, which can be translated into an epidemiological Odds Ratio:

$$OR_i = e^{\beta_i}. \tag{S.4}$$

S.2.2. Parameter Estimation and Ridge Regularization

Model parameters (the coefficients β_i) were estimated by Maximum Likelihood Estimation (MLE) (Hosmer Jr. et al., 2013). To prevent the aggressive elimination of correlated biological

variables (e.g., T and T^2, where T is temperature), models were penalized exclusively using Ridge ($L2$) regularization. The Ridge-penalized objective is:

$$\mathcal{L}_{\text{Ridge}}(\boldsymbol{\beta}) = -\ell(\boldsymbol{\beta}) + \frac{1}{C}\sum_{i=1}^{n}\beta_i^2, \tag{S.5}$$

where $\ell(\boldsymbol{\beta})$ is the log-likelihood and C is the inverse regularization strength. Unlike Lasso ($L1$) regularization, which arbitrarily zeroes out correlated features, Ridge smoothly distributes weights across correlated predictors, preserving epidemiologically meaningful covariates that would otherwise be discarded.

S.2.3. Three-Step Modeling Pipeline

The three nested models referenced in Section 2.5 of the main manuscript were implemented as `scikit-learn` pipelines as follows:

1. Baseline Linear Model (BLM):
   `Pipeline([StandardScaler, SelectKBest, LogisticRegression])` with the intercept constrained to zero. Hyperparameters were tuned via `GridSearchCV` over the k value of `SelectKBest` and the inverse regularization strength C.
2. Fully Engineered Model (FEM):
   `PolynomialFeatures(degree=2), LogisticRegression])` with the intercept constrained to zero. No feature selection was applied; only the Ridge penalty C was tuned.
3. Enhanced Fully Engineered Model (FEM+):
   `Pipeline([StandardScaler, PolynomialFeatures(degree=2), RFE(LogisticRegression), LogisticRegression])` with the intercept constrained to zero. `GridSearchCV` jointly tuned the inverse regularization strength C and the `n_features_to_select` parameter of `RFE`.

All pipelines used 5-fold cross-validation on the training set (70% of paired samples) for hyperparameter tuning, with final performance evaluated on the held out test set (30%).

S.2.4. Software Environment

All analyses were conducted in Python, using `pandas` (version 2.2.3) for data manipulation, `numpy` (version 2.2.5) for numerical operations, `scikit-learn` (version 1.7.1) for machine learning, `statsmodels` for statistical analysis, and `matplotlib` (version 3.10.7) and `geopandas` (version 1.1.1) for visualization.

REFERENCES

Hosmer Jr., D. W., Lemeshow, S., & Sturdivant, R. X. (2013). *Applied Logistic Regression*. Hoboken: John Wiley & Sons, Ltd. doi:https://doi.org/10.1002/9781118548387.fmatter.
